# Diagnosis of Diphtheria

**Published:** 1893-12

**Authors:** 


DIAGNOSIS OF DIPHTHERIA.—Every physician knows both the importance and the difficulty in diagnosing a case of diphtheria. In New York there have been established free dispensaries, under the charge of expert bacteriologists, paid by the city, for the purpose of assisting practitioners in a ready diagnosis. In a doubtful case a specimen of the exudate can be sent to one of these dispensaries for examination, and within twenty-four hours the doctor can learn whether or not it contains the dreaded Loffler bacillus. Just what one is going to do in those cases of masked diphtheria,—where the disease does not come in sight, as it were, and hence no specimen can be had,—deponent sayeth not. Apropos: In our last our intelligent compositor made a *faux-pas.* We wrote, "The homeopaths, according to their figures and diagnoses, cure more diphtheria than all the regular doctors put together see"; meaning that the homeopaths diagnose all sore throat diphtheria. The printer spoiled the point by putting an interrogation point after "see," thus: "See?" (Confound the printer.)

